# Recurrent Primary Extracranial Meningioma: A Case Report and Literature Review of a Rare Scalp Tumor

**DOI:** 10.7759/cureus.103281

**Published:** 2026-02-09

**Authors:** Renad Alduwayan, Hydar AlQassab, Hisham Alkhalidi, Hana Elwatidy, Ziyad Alharbi, Hesham Alshehri, Sherif Elwatidy

**Affiliations:** 1 Medicine, King Khalid University Hospital (KKUH), Riyadh, SAU; 2 Pathology, King Saud University College of Medicine, Riyadh, SAU; 3 Neurological Surgery, Newgiza University, Cairo, EGY; 4 Neurological Surgery, King Khalid University Hospital (KKUH), Riyadh, SAU

**Keywords:** extracranial meningioma, immunohistopathology, meningioma, radiological characteristics, scalp tumors

## Abstract

Extracranial meningiomas are rare tumors that arise outside the cranial cavity and can clinically mimic benign soft tissue lesions. Due to their slow growth and nonspecific appearance, they are often misdiagnosed. Complete surgical excision remains the mainstay of treatment. A 35-year-old female presented with a three-year history of a painless, slowly growing left frontal scalp swelling. On examination, the lesion measured approximately 3-4 cm in diameter, was firm, had normal overlying skin, was very sensitive to touch, and showed no neurological deficits. Surgical excision was performed under general anesthesia. A well-defined lesion was identified and completely excised. At follow-up, the patient developed a recurrent small tumor after seven months and was closely observed. Primary extracranial meningiomas (PEMs) are rare tumors that may involve the scalp. The origin and pathogenesis of PEMs remain theoretical; awareness of their imaging and pathological characteristics is essential for accurate diagnosis. Surgical excision is the primary treatment modality, with outcomes depending on tumor characteristics and completeness of resection.

## Introduction

Meningiomas are typically benign tumors that arise from the meningeal layers of the brain or spinal cord and account for approximately 37.6% of all primary central nervous system tumors [1]. Compared with intracranial meningioma, extracranial meningiomas are rare. Studies from large centers report that extracranial meningiomas represent about 1-2% of all meningiomas [2]. Proper classification is important, especially when determining tumor origin. Extracranial meningiomas are classified as primary or secondary, with secondary tumors - arising from direct extension of an intracranial meningioma - being the most common [3]. Primary extracranial meningiomas (PEMs) are rare and are thought to originate from ectopic arachnoid cells or postoperative seeding. They most commonly involve the head and neck region, with reported locations including the scalp, ear and earlobe, nasal cavity, paranasal sinuses, orbit, parotid gland, and cervical soft tissues [4]. The clinical presentation of PEMs is often subtle, with localizing signs or symptoms typically appearing only after the tumor reaches a considerable size. Clinical manifestations are mainly due to local mass effect and neurological dysfunction resulting from involvement of adjacent cranial nerves. Prognosis is variable but is often favorable following adequate surgical management.

## Case presentation

**Figure 1:**
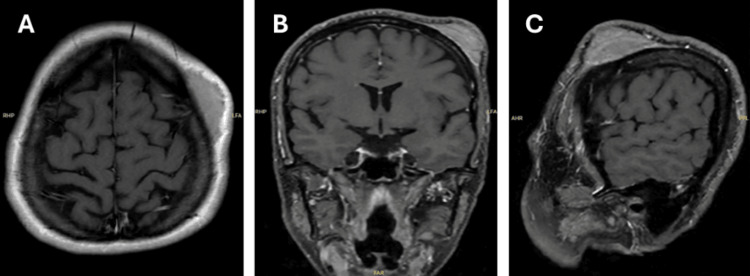
(A-C) Preoperative MRI T1 images after gadolinium injection (axial, coronal, and sagittal, respectively) showing the scalp lesion. Note: No bone or dural enhancement.

A 35-year-old woman with a known history of hypothyroidism presented with a three-year history of progressively enlarging, asymptomatic swelling in the left frontal scalp region. For the past six months, she reported hypersensitive skin over the swelling and persistent discomfort. She denied any neurologic symptoms suggestive of an intracranial lesion, such as diplopia, facial numbness, visual changes, seizures, or constitutional symptoms. On examination, there was a firm mass measuring 2-3 cm, with no signs of infection, including discharge or redness, and no neurological deficits. The skin over the swelling was very sensitive to touch, and the patient was unable to tolerate examination. No cervical lymphadenopathy was noted. MRI with and without contrast showed a well-circumscribed, enhancing, homogeneous, benign lesion with normal-appearing cortical and subcortical structures (Figures 1A-1C).

**Figure 2:**
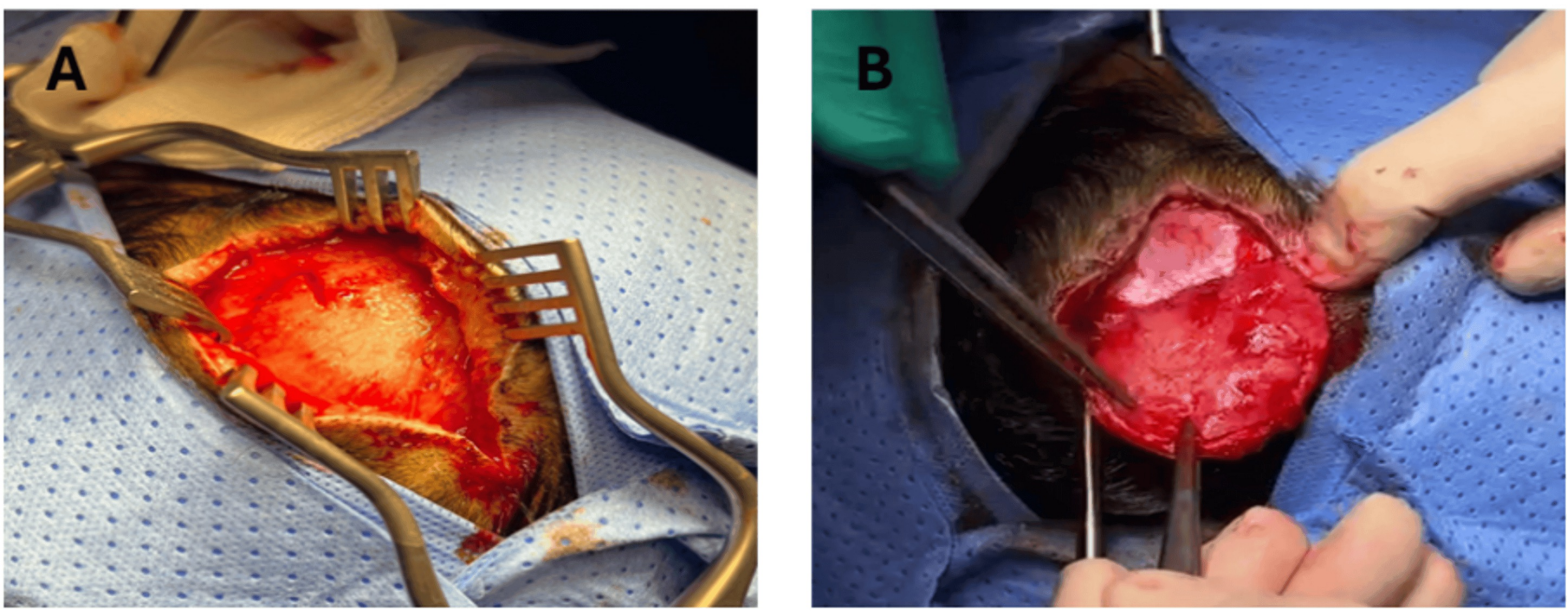
(A) Intraoperative image after exposure of the tumor. (B) Intraoperative image after en-bloc dissection of the tumor together with the pericranium. Note: Healthy bone beneath the tumor.

The patient underwent excision of the left frontal scalp swelling under general anesthesia. Dissection was carried out through the subcutaneous tissue, and the lesion was carefully exposed along with the surrounding pericranium. En bloc excision of the lesion, together with 3-4 mm of the surrounding pericranium, was performed using monopolar cautery. Histopathological examination confirmed a meningothelial meningioma, WHO grade I, with no atypical features. Molecular analysis, according to the WHO classification, revealed no high-risk molecular alterations. There were no bony changes under the lesion. The procedure was completed without complications (Figures 2A-2B).

The patient recovered well postoperatively and had no complications. During her follow-up in the outpatient department, she noticed a recurrence of a small lesion at the same site. On examination, there was a small 3-4 mm mass with the same features as the primary lesion. Imaging obtained at that time revealed a recurrent soft-tissue lesion at the previous operative site. The case was subsequently discussed at the tumor board, where the decision was to continue close radiological surveillance and to plan surgical intervention (remove the tumor with the underlying bone and dura) if the tumor continues to grow on follow-up imaging or if the patient develops new or worsening symptoms.

## Discussion

Unlike primary intracranial meningiomas (PIMs), PEMs are a rare type of meningiomas that develop outside the cranial cavity, usually in the head and neck region, and accounts for approximately 1-2% of all reported meningioma cases [5,6]. PEMs are often misdiagnosed as other soft tissue tumors, such as schwannomas, paragangliomas, or fibrous histiocytomas, highlighting the importance of histopathological and immunohistochemical evaluation. Epidemiologically, PEMs show a bimodal age distribution, with incidence peaks in the second decade of life and again between the fifth and seventh decades. In contrast to intracranial meningiomas, which have a pronounced female predominance, extracranial meningiomas show a more variable sex distribution. Some series report an approximately equal male-to-female ratio, while others show a mild female predominance that is most likely to be influenced by the tumor's anatomical location [7].

Extracranial meningiomas are usually classified according to the system proposed by Hoye et al., which categorizes these lesions based on their relationship to an intracranial meningioma. In this system, extracranial meningiomas are divided into primary (ectopic) and secondary subtypes. Secondary extracranial meningiomas come from direct extension of an intracranial tumor or, less commonly, metastatic spread, while PEMs develop independently without intracranial, dural, or foraminal connection. Several pathogenetic mechanisms have been used to explain the development of PEMs, including embryonic displacement of arachnoid cap cells, migration of meningothelial cells along cranial nerve sheaths, and mesenchymal metaplasia of local tissues. These hypotheses were used as explanatory models rather than formal classification systems [4,6].

In our case, the frontal scalp lesion showed no radiological or intraoperative evidence of intracranial, dural, or osseous invasion, supporting its classification as a primary extracranial (ectopic) meningioma. The diagnosis was confirmed based on histopathological features, including meningothelial cell morphology, and immunohistochemical positivity for epithelial membrane antigen (EMA) and vimentin, with a low Ki-67 proliferative index. Given the rarity of these tumors, the main differential diagnoses included schwannomas, fibrous histiocytomas, paragangliomas, and other benign or malignant soft tissue neoplasms, which were excluded based on histopathology and immunoprofile. Clinically, PEMs typically have an indolent presentation. Most patients present with a slowly enlarging, painless scalp or soft-tissue mass, and focal neurological symptoms are uncommon unless there is secondary intracranial extension or involvement of adjacent neurovascular structures. When symptoms occur, they are generally related to local mass effects, such as discomfort, tenderness, or cutaneous hypersensitivity, rather than true neurological deficits. Consistent with this presentation, our patient complained of a gradually enlarging left frontal scalp mass over a three-year period, which was initially asymptomatic and later associated with localized hypersensitivity and persistent discomfort, without any neurological or constitutional symptoms.

**Figure 3:**
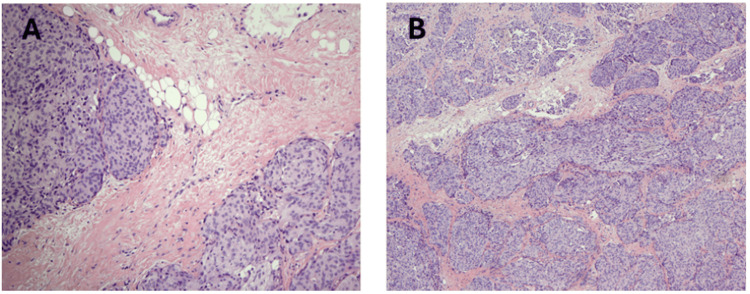
(A) Microscopic examination of the scalp tissue reveals a tumor composed of lobulated nests and syncytial arrangements of meningothelial cells infiltrating the subcutaneous tissues (H&E, X40). (B) At higher magnification (H&E, X100), the characteristic meningothelial cells display oval nuclei, delicate chromatin, and indistinct cell borders, forming occasional vague whorls within a fibrocollagenous stroma.

**Figure 4:**
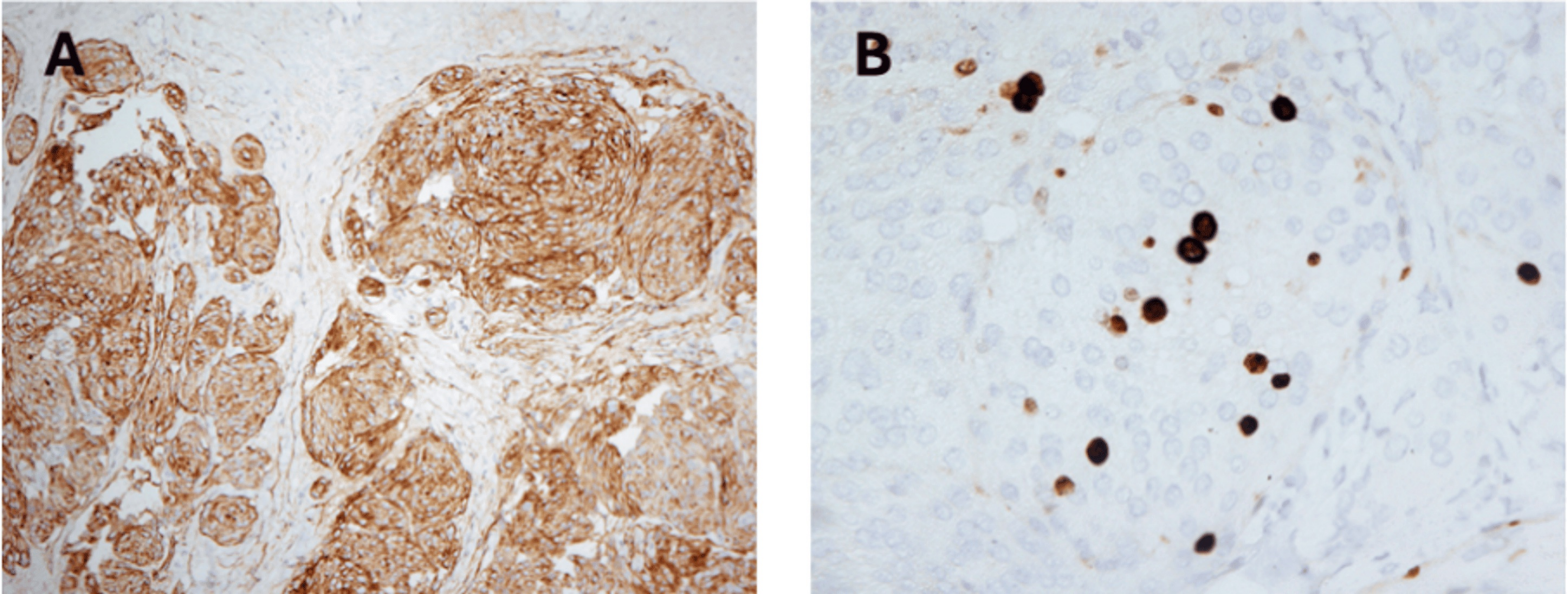
(A) Immunohistochemical study for epithelial membrane antigen (EMA) showed strong, diffuse membranous and cytoplasmic reactivity within the tumor cell nests, supporting the diagnosis of meningioma (EMA, X100). The Ki-67 proliferative index is focally increased, estimated at approximately 8-10% in this high-power field, as indicated by positive nuclear staining in a subset of tumor cells (Ki-67, X400).

Radiologically, PEMs typically appear on MRI, as well-circumscribed extracranial soft-tissue masses that show homogeneous enhancement after gadolinium administration, without intracranial extension, dural tail sign, or calvarial involvement. In our case, contrast-enhanced T1-weighted MRI in axial, coronal, and sagittal planes (Figure 1) revealed a well-enhancing scalp lesion without evidence of bone erosion or dural enhancement, findings characteristic of a PEMs. Histologically, PEMs share the same microscopic and immunohistochemical features as their intracranial counterparts (Figure 3). These tumors are composed of meningothelial cells and typically show immunoreactivity for EMA (Figure 4A) and vimentin, while S-100 protein expression may be variable, and glial fibrillary acidic protein (GFAP) is usually negative. Despite their extracranial location, PEMs generally display benign histologic characteristics and lack true malignant behavior, as supported by a low proliferative index, with Ki-67 (Figure 4B) values most commonly reported below 5%. The histogenesis of PEMs is thought to involve ectopic arachnoid cells, meningeal derivatives, or mesenchymal cells undergoing secondary meningothelial differentiation [8,9]. 

Surgical excision remains the gold standard treatment for extracranial meningiomas and should be carefully planned using preoperative imaging to determine tumor extent and to exclude an associated intracranial meningioma. The surgical approach differs between primary and secondary extracranial meningiomas. PEMs should be managed as conventional soft-tissue tumors, with the primary goal of complete surgical excision. In contrast, secondary extracranial meningiomas are extensions of intracranial lesions, often spreading through cranial foramina or eroding the skull base and frequently involving critical neurovascular structures. In these cases, management typically requires a combined approach, beginning with craniotomy and resection of the intracranial component, followed by a transfacial or extracranial approach to address the extracranial portion. The extent of surgical intervention depends on the degree of bone erosion, tumor size, and infiltration of surrounding tissues [10]. To contextualize our case within the existing literature, Table 1 summarizes previously reported cases of PEMs confined to the scalp, adapted from the systematic review by Umana et al. [11]. From that review, only scalp-localized cases were extracted to ensure direct relevance to the present case. The summarized data indicate that scalp PEMs most commonly present as solitary lesions with meningothelial histology and are primarily managed by complete surgical excision, with generally favorable outcomes. Recurrence appears uncommon following total resection, although isolated recurrent cases have been described [10]. In some reports, recurrent lesions were managed with repeat surgical excision, and a few cases received adjuvant radiotherapy, particularly when complete resection was not feasible. Complications in these cases were generally minimal, but these reports highlight the importance of achieving adequate surgical margins and ensuring long-term follow-up. Overall, the scalp-specific findings reported in the literature are consistent with the indolent clinical course and favorable outcome observed in our patient [11].

**Table 1:** Summary of scalp cases of primary extracranial meningiomas. Adapted from the systematic review by Umana et al. [11]; only cases localized to the scalp were included to align with the present report.

Authors and Year	Patients (n)	Age/Sex	Localization	Histology and Grading	Treatment	Recurrence	Outcome
Miyamoto et al., 1995 [12]	1	15 F, 13 M	2 Occipital scalp	1 Meningothelial, 1 Fibroblastic	Surgical resection	No recurrence	Complete recovery
Shaw et al., 2004 [13]	2	29 M	Occipital scalp	Meningothelial meningioma	Surgical resection	No recurrence at 12 months	Complete recovery
Eshete et al., 2005 [14]	3	70 M	Parietal scalp	Meningothelial meningioma	Surgical resection	No recurrence	Not available
Alduwayan et al., 2026 (current case)	4	1 F	Frontal scalp	Meningothelial meningioma	Surgical resection	Yes, there's recurrence	Tecurrence

## Conclusions

Primary extracranial meningiomas are rare tumors often overlooked in the differential diagnosis of head and neck soft tissue masses. Although the origin and pathogenesis of PEMs remain theoretical, awareness of their imaging and pathological characteristics is essential for accurate diagnosis. Complete surgical excision provides excellent long-term outcomes, although isolated cases of recurrence have been reported, highlighting the importance of adequate surgical margins and long-term follow-up.

## References

[1] Ostrom QT, Cioffi G, Gittleman H, Patil N, Waite K, Kruchko C, Barnholtz-Sloan JS (2019). CBTRUS statistical report: primary brain and other central nervous system tumors diagnosed in the United States in 2012-2016. Neuro Oncol.

[2] Whicker JH, Devine KD, MacCarty CS (1973). Diagnostic and therapeutic problems in extracranial meningiomas. Am J Surg.

[3] Farr HW, Gray GF Jr, Vrana M, Panio M (1973). Extracranial meningioma. J Surg Oncol.

[4] Hoye SJ, Hoar CS Jr, Murray JE (1960). Extracranial meningioma presenting as a tumor of the neck. Am J Surg.

[5] Bassiouni H, Asgari S, Hübschen U, König HJ, Stolke D (2006). Dural involvement in primary extradural meningiomas of the cranial vault. J Neurosurg.

[6] Lang FF, Macdonald OK, Fuller GN, DeMonte F (2000). Primary extradural meningiomas: a report on nine cases and review of the literature from the era of computerized tomography scanning. J Neurosurg.

[7] Goh XL, Chee JR, Tham AC (2025). Primary extracranial meningiomas of the sinonasal tract: a systematic review. J Neurol Surg B Skull Base.

[8] Shuangshoti S, Panyathanya R (1973). Ectopic meningiomas. Arch Otolaryngol.

[9] Kepes JJ (1982). Meningiomas: Biology, Pathology, and Differential Diagnosis. https://searchworks.stanford.edu/view/L12425.

[10] Iaconetta G, Santella A, Friscia M, Abbate V, Califano L (2012). Extracranial primary and secondary meningiomas. Int J Oral Maxillofac Surg.

[11] Umana GE, Scalia G, Vats A (2021). Primary extracranial meningiomas of the head and neck. Life (Basel).

[12] Miyamoto T, Mihara M, Hagari Y, Shimao S (1995). Primary cutaneous meningioma on the scalp: report of two siblings. J Dermatol.

[13] Shaw R, Kissun D, Boyle M, Triantafyllou A (2004). Primary meningioma of the scalp as a late complication of skull fracture: case report and literature review. Int J Oral Maxillofac Surg.

[14] Eshete M, Schneider J (2005). Extracranial meningioma of the scalp: case report. Ethiop Med J.

